# Prevalence of Cognitive Impairment and Its Predictors among HIV/AIDS Patients on Antiretroviral Therapy in Jimma University Medical Center, Southwest Ethiopia

**DOI:** 10.1155/2019/8306823

**Published:** 2019-03-13

**Authors:** Getachew Yideg Yitbarek, Andualem Mossie Ayana, Moyeta Bariso Gare, Gashaw Garedew Woldeamanuel

**Affiliations:** ^1^Department of Biomedical Sciences, College of Health Sciences, Debre Tabor University, Debre Tabor, Ethiopia; ^2^Department of Biomedical Sciences, College of Health Sciences, Jimma University, Jimma, Ethiopia; ^3^Department of Biomedical Sciences, College of Medicine and Health Science, Wolkite University, Wolkite, Ethiopia

## Abstract

**Background:**

Cognitive impairment among human immunodeficiency virus (HIV) infected patients can lead to treatment nonadherence, faster progression of the illness, disability, and bed ridden state if we fail to detect it early. However, there is scarcity of previous published studies in Ethiopia on the assessment of cognitive impairment among HIV-positive patients. Hence, this study aimed to determine the prevalence and associated factors of cognitive impairment among HIV-positive patients receiving antiretroviral therapy (ART) at Jimma University Medical Center, Ethiopia.

**Methods:**

Hospital-based cross-sectional study was conducted among 328 HIV-positive patients attending Jimma University Medical Center, Ethiopia. Data were collected from a face-to-face interview and review of medical records using semistructured questionnaire. Validated International HIV Dementia Scale (IHDS) was used to screen for cognitive impairment. Data was analyzed using SPSS version 20.

**Results:**

A total of 328 (191 females and 137 males) HIV-positive patients were included in the study with a response rate of 97.04%. The prevalence of cognitive impairment among HIV-positive patients was 35.7%. Factors significantly associated with cognitive impairment were age group of 41−64 years (adjusted odds ratio [AOR] = 3.1, 95% confidence interval [CI] (1.3, 7.4)], plasma HIV-1 RNA load between 1.7log10 and 3log10 copies/ml [AOR = 2.2, 95% CI (1.1,4.3)] and ≥ 3log10 copies/ml [AOR = 7.5, 95% CI (2.6, 21.5)], khat chewing [AOR = 4.4, 95% CI (2.3, 8.3)], and clinical stage III of the disease [AOR = 5.6, 95% CI (1.7, 19.2)].

**Conclusion:**

Despite the use of ART, the burden of cognitive impairment among HIV patients was high. Older age, khat chewing, advanced stage of the disease, and higher viral load were the independent factors associated with cognitive impairment. Thus, continuous screening of cognitive impairment, identification of the possible risk factors, and proper management strategy should be designed.

## 1. Introduction 

HIV has emerged as a major threat to world health since the start of the epidemic and has challenged scientists and clinicians to combat its vast and devastating effects that led to the death of 1.5 million people from acquired immunodeficiency syndrome (AIDS) related illnesses in 2015 alone [1].

Although the introduction of ART drugs has prolonged the survival of HIV-positive patients, neurological complications of the disease have remained a great source of morbidity, which can also lead to treatment nonadherence, faster progression of the illness, and disability [2, 3]. Failure to recognize cognitive impairment will harm the efforts to control HIV in a community as cognitively impaired patients are more likely to engage in HIV-related risk activity [4].

The nature of HIV-related cognitive impairment ranges from the asymptomatic to mild forms and to the more severe form. A report in the multicenter international studies revealed that the cognitive impairment among HIV-positive people ranged from 13% in Brazil to 18.4% in Thailand and from 34% in China to 56% in India [5]. Africa shoulders 75% of the world's burden of AIDS related deaths, which was mainly due to HIV-associated neurocognitive impairment [6]. A study conducted in resource-limited countries in 2009 shows that the problem of neurocognitive impairment in sub-Saharan area ranges from 20% to 37% [5]. A study conducted in Northern Nigeria, Malawi, Botswana, and Kenya also showed that the prevalence of cognitive impairment was 21.5%, 14%, 38%, and 15.3%, respectively [3, 4, 7, 8]. In Ethiopia, a study conducted among HIV-positive participants showed that the prevalence of HIV-associated dementia was 33.3% [7].

There is evidence that cognitive functioning in HIV is associated with disease severity (CD4 T-cell count level less than 200 cell/*μ*l and plasma HIV-1 RNA load), advanced age, level of education, income level, social support, medical comorbidities, and HIV medication adherence [9–16]. Previous study showed that khat chewing declines memory and learning, resulting in cognitive impairment [16, 17]. Moreover, age-related dementia usually happens at the age of 65 years and above [7].

Different studies investigated cognitive impairment among HIV patients worldwide but there is scarcity of such studies in Ethiopia. Above all, the impacts of body composition, cohypertension, and substance use on cognitive impairment among HIV-positive patients have not been clearly elucidated. Hence, the aim of this study is to determine the prevalence and associated factors of cognitive impairment among HIV-positive patients receiving ART.

## 2. Materials and Methods

### 2.1. Study Area and Period

The study was conducted at ART clinic of Jimma University Medical Center (JUMC), Jimma, Ethiopia. The hospital is in Jimma town, which is located 352 kilometers southwest of Addis Ababa, Ethiopia. JUMC is providing services for approximately 15 million people in the catchment area including ART service for HIV-positive patients coming from different areas. In the year 2018, about 6090 HIV patients were registered in the clinic. Of these, 3116 had active follow-up and the rest of patients have been reported as lost to follow-up, transfer-out, and death. All patients who had active follow-up were on ART. Institution-based cross-sectional study was conducted from April 7 to May 7, 2018, at ART clinic of JUMC.

### 2.2. Population and Eligibility Criteria

The source population of this study was all HIV-positive patients enrolled to JUMC ART clinic. Samples of HIV-positive patients attending ART clinic of JUMC during the study period were the study population. The inclusion criteria for this study were age group of 18-64 years (because age-related neurocognitive impairments are common above the age of 65 years) and those who had complete medical records. Those with known cognitive impairment unrelated to HIV (psychiatric disorder, severe medical illnesses, and previous history of stroke) and those clients who had visual, hearing, and speech difficulty were excluded from the current study.

### 2.3. Sample Size and Sampling Techniques

The actual sample size was determined by using the single population proportion formula, where the following assumptions were considered: 95% confidence interval, 33.3% proportion of neurocognitive impairment among HIV-positive patients [7], and 5% margin of error. Since the total population was 3116, we employed a correction formula, and then 10% nonresponse rate was added, which gave rise to a final sample size of 338.* The participants were selected through systematic random sampling technique* after having the monthly client flow to the hospital.

### 2.4. Operational Definitions

HIV-associated cognitive impairment was defined as any HIV-positive patient with a score of 10 or less on IHDS [18]. Lifetime substance use (khat, cigarette, and alcohol) refers to any participant who had history of consumption of any of the substance (khat, cigarette, and alcohol) for more than two years.

### 2.5. Data Collection Procedures

A total of four data collectors (two psychiatric nurses and two ART nurses) were employed to collect the data. Data were collected through face-to-face interview using semistructured questionnaire and review of medical records for HIV-related clinical factors. IHDS, which was translated into local language (Amharic and Afaan Oromoo), was used to screen for cognitive impairment. The IHDS has been a useful screening test to identify individuals at risk for HIV-associated cognitive impairment. It consists of three subtests: timed finger tapping, timed alternating hand sequence test, and recall of four items at 2 minutes. A total score out of 12 was calculated for each participant, and any participant with a score of 10 or less was screened as at risk for cognitive impairment; hence, they were recommended to be evaluated further for possible cognitive impairment [18–20]. Finally, the data collectors have measured the weight (to the nearest 0.1kg), height (to the nearest 0.1cm), hip and waist circumferences (to the nearest 0.1centimeter), and BP (to the nearest 0.5mmHg) of the participants whose data were kept anonymous. Body mass index (BMI) and waist to hip ratio of the participants were then calculated.

### 2.6. Data Quality Control

The questionnaire was translated from English to local language (Amharic and Afaan Oromoo) and then retranslated back to English to maintain its consistency. Training was provided for the data collectors before the actual data collection. Pretest was done among 17 HIV-positive patients at Shenen Gibe Primary Hospital in order to see the validity of the instrument to estimate the time needed to collect data, and the questionnaires were modified accordingly. Data collectors were supervised in daily fashion and the data was checked for completeness and consistency throughout the data collection period.

### 2.7. Data Analysis

Data were edited, coded, and entered into Epi Data version 3.1 and then exported to SPSS version 20 for analysis. Descriptive statistics like frequencies and percentages were used to present sociodemographic characteristics. Odds ratio (OR) and confident interval (CI) were used to determine the strength of association between dependent and independent variables. Binary logistic regression analysis was performed to rank the relative importance of exposure variables with outcome variables. The variables that have statistically significant (p ≤ 0.25) associations with the outcome variable in the binary logistic regression analysis were further considered as a candidate for backward stepwise multiple logistic regression model to control the effect of confounding variables. Finally, those variables with p value less than 0.05 in the final model were considered as statistically significant.

### 2.8. Ethical Consideration

Ethical clearance was obtained from Institutional Review Board of Jimma University, Ethiopia, with ethical approval reference number IHRPGD/252/18 and letter of permission to conduct the study was obtained from JUMC clinical director office. Each participant was then informed about the purpose of the study and his/her right not to participate in the study was respected. Privacy and confidentiality were assured. Data was collected after obtaining written informed consent from the participants.

## 3. Results

### 3.1. Sociodemographic Characteristics of Study Participants

A total of 328 HIV-positive patients who were attending Jimma University Medical Center participated in the study with a response rate of 97.04%. Majority (191 (58.2%)) of the respondents were females and the mean age (± SD) of the participants was 38.22 (±10.5) years with the age range between 18 and 64 years. Regarding the educational status, majority (148 (45.1%)) of the participants had less than grade 8 educational level. About 174 (53%) of the participants were married and 113 (34.5%) of the participants had a monthly income of ≥1500 Ethiopian Birr/month (Table 1).

### 3.2. Substance Use and Anthropometric Characteristics of the Participants

Regarding substance use, 113 (34.5%) of the participants had history of khat chewing in their lifetime, while 45 (13.7%) of them had consumed alcohol in their lifetime. Waist to hip ratio measurement among study participants showed that 176 (92.1%) of female participants scored greater than 0.8 and 117 (85.4%) of male participants scored below 0.9. Among the total participants, 221 (67.4%) had BMI values between 18.5 kg/m^2^ and 24.9 kg /m^2^ (Table 2).

### 3.3. Clinical Characteristics of the Study Participants

The mean duration of the illness since diagnosis was 5.5 years with 257 (78.4%) of the participants having the illness beyond two years of duration. Out of the total participants, about 265 (80.8%) were on follow-up for ≥5 years and 105 (32%) of the participants had CD4 T-cell count greater than 500 cells/*μ*l. Plasma HIV-1 RNA load status of the participants showed that 174 (53%) of the participants had plasma HIV-1 RNA load less than 1.7log10 copies/ml (Table 3).

### 3.4. Prevalence of Cognitive Impairment

Among the study participants, 117 scored 10 or less on IHDS. Thus, the prevalence of HIV-associated cognitive impairment was 35.7% (95% CI, 30.8%-40.9%). Regarding timed finger tapping, motor speed was assessed and 179 (54.6%) did well, scoring 4/4. However, 17 (5.2%) and 2 (0.6%) of the participants did worse, scoring 2/4 and 1/4, respectively. Regarding psychomotor speed, 133 (40.5%) of them worked well without any impairment, scoring 4/4, and 22 (6.7%) of them scored 2/4. Assessment of memory recall also showed that 191 (58.2%) of the participants recalled all the four things without any clue, scoring 4/4, but about 15 (4.5%) of the participants scored less than 2.5/4 even with a clue. Overall, 211 (64.3%) of the participants had no cognitive impairment.

### 3.5. Factors Associated with Cognitive Impairment

All variables that had p value ≤ 0.25 in the univariable analysis were included in the multivariable analysis. After adjusting for these variables, age category of 4–64 years [AOR = 3.1, 95% CI (1.3, 7.4), p = 0.01], WHO clinical stage III and above [AOR = 5.6, 95% CI (1.7, 19.2), p = 0.01], chewing khat for more than 2 years [AOR = 4.4, 95% CI (2.3, 8.3), p =0.001], plasma HIV-1 RNA load between 1.7log10 and 3log10 copies/ml [AOR=2.2, 95% CI (1.1,4.3), p=0.02], and greater than 3log10 copies/ml [AOR = 7.5, 95% CI (2.6, 21.5), p = 0.001] were significantly associated with cognitive impairment (Table 4).

## 4. Discussion

The present study showed that the prevalence of cognitive impairment was 35.7%. This finding is comparable with previous studies conducted in Ethiopia, Uganda, and China [15, 18, 21], which showed prevalence of cognitive impairment as 33.3%, 31%, and 34%, respectively. However, it is less marked than the findings of other studies conducted in India, USA, and Italy [15, 18, 19] which reported a prevalence rate of 56%, 49%, and 55%, respectively. The higher prevalence reported in those studies might be due to the difference in rating scales along with the variance in sociodemographic characteristics.

The prevalence of cognitive impairment among HIV-positive patients reported in the present study is very high compared to the report of multicenter international studies that revealed neuropsychological deficit among HIV-positive patients as 13% in Brazil and 18.4% in Thailand [5]. The prevalence of cognitive impairment reported in this study is also higher than the findings of other studies conducted in Malawi (14%) and Kenya (15.3%) [15, 16] which showed that cognitive impairment occurred in 14% and 15.3% of the study populations, respectively. Different viral clades and screening tools used may account for the variation in the magnitude of neurocognitive impairment as certain clades may be more or less neuropathogenic. Several HIV clade subtypes have evolved with distinct genetic differences that follow geographic boundaries. Subtypes A and D strains are predominant in Kenya, while subtype C strain is common in Ethiopia, and HIV-associated cognitive impairment has been observed to be relatively higher in clade types B and C HIV infection [18, 22].

The present study revealed that older age (age group of 41 years or more) was significantly associated with development of HIV-associated cognitive impairment, which is in agreement with studies conducted in Ethiopia and Malawi [9, 16]. The possible reason might be accounted to poor response to ART and faster progression of the disease at old age.

The present study showed that khat chewing has been significantly associated with cognitive impairment, which is in line with previous studies conducted in Netherlands and Kenya [16, 17]. Considering the similarity between cathinone and amphetamine, previous studies in humans have shown that psychoactive drugs increase concentration of neurotransmitters such as dopamine, serotonin, and noradrenaline in specific regions of the brain, which results into an influx of neurotransmitter at the synaptic cleft. Therefore, excessive stimulation of dopaminergic neurons by cathinone, the active ingredient of khat, could have led to damage and inhibition of dopamine uptake at the receptors in a dose-dependent manner and hence no interneuronal communication that could have resulted in impaired working memory [23]. Decreased neurotransmitters uptake, particularly in the striatum due to damage to receptors by repeated exposure to cathinone, had been found to be associated with impairment of the updating rather than the maintenance component of working memory. Khat users are less efficient in discriminating which information is relevant, so that nonrelevant information is more likely to enter WM for storage which interferes with memory [17].

In agreement with the study conducted in Nigeria [9], the present study showed that high detectable plasma HIV-1 RNA load while on ART is significant predictor of cognitive impairment. This is due to the fact that, over the course of the disease, higher plasma HIV-1 RNA load correlates with the degree of CNS dysfunction by overwhelming host defenses (the blood brain barrier), brain plasticity, and cognitive reserves, which further result in cognitive impairment [21, 23].

The present study also revealed that late clinical stage was a significant predictor for the development of cognitive impairment. This finding is supported by other studies conducted in Ethiopia and Cameroon, where advanced HIV diseases were risk factors for cognitive decline [24]. It was believed that advancement of the disease is reported more in clients with late stage of the disease [25]. As a result, the complications associated with the advancement of the disease and the drugs used to control those complications may account for the development of cognitive impairment.

### 4.1. Limitations of the Study

The present study had some limitations. This study was a cross-sectional study that we could not establish causal relationships between the associations we observed. Moreover, the IHDS, which is validated in sub-Saharan countries, was not yet validated among HIV patients receiving ART in Ethiopia [18].

## 5. Conclusions

The prevalence of cognitive impairment among HIV patients receiving ART was high, where nearly one-third of the study participants had cognitive impairment. This is a major public health problem that needs attention of researchers, physicians, and policy-makers. The present study also indicated that age group of 41 years or more and HIV-1 RNA load level between 1.7log10 and 3log10 and greater than 3log10 copies/ml, being in late stages of the disease, and khat chewing were found to be a risk factor for cognitive impairment. Therefore, continuous screening of cognitive impairment, identification of the possible risk factors, and proper management strategy should be designed. Additionally, as cognitive status may be a marker for health status and quality of life, longitudinal study would be needed to verify cause-effect relationship of cognitive impairment and presumed risk factors.

## Figures and Tables

**Table 1:** Sociodemographic characteristics of HIV-positive patients attending Jimma University Medical Center, Jimma, Ethiopia, 2018 (N= 328).

Characteristics		Frequency	Percentage
Age (years)	18-30	90	27.5
	31-40	127	38.7
	41-64	111	33.8

Sex	Female	191	58.2
	Male	137	41.8

Educational status	< grade 8	148	45.1
	Grade 9-12	101	30.8
	Diploma and above	79	24.1

Marital status	Single	50	15.2
	Married	174	53.1
	Divorced	50	15.2
	Widowed	54	16.5

Occupation	Government employed	107	32.6
	Merchant	66	20.1
	House wife	17	5.2
	Daily laborer	36	11
	Others *∗*	29	8.8

Monthly income (ETB/month)	<500	68	20.7
	500-1000	97	29.6
	1001-1500	50	15.2
	≥1500	113	34.5

Residence	Urban	286	87.2
	Rural	42	12.8

*Note*. Others*∗* = students, unemployed; ETB = Ethiopian Birr.

**Table 2:** Substance use and anthropometric characteristics of HIV-positive patients attending Jimma University Medical Center, Jimma, Ethiopia, 2018 (N = 328).

Characteristics			Frequency	Percentage (%)
Lifetime khat chewing		Yes	113	34.5
		No	215	65.5

Current khat chewing		Yes	88	26.8
		No	240	73.2

Lifetime alcohol drink		Yes	45	13.7
		No	283	86.3

Current alcohol drink		Yes	14	4.3
		No	314	95.7

BMI (kg/m^2^)		18.5-24.9	221	67.4
		<18.5	52	15.9
		≥25	55	16.7

WHR	Female	<0.8	15	7.9
		≥0.8	176	92.1
	Male	<0.9	117	85.4
		≥0.9	20	14.6

*Note*. BMI = body mass index; WHR = waist to hip ratio.

**Table 3:** Clinical characteristics of HIV-positive patients attending JUMC, Jimma, Ethiopia, 2018 (N = 328).

Clinical factors		Frequency	Percentage (%)
Comorbid hypertension	Yes	75	22.9
	No	253	77.1

Duration of illness since diagnosis (years)	<2	71	21.6
	≥2	257	78.4

CD4 T-cell count (cell/*μ*l)	<200	80	24.4
	200-350	55	16.8
	351-500	88	26.8
	≥500	105	32.0

Plasma HIV-1 RNA load (copies/ml)	<1.7log10	174	53
	1.7log10-3log10	97	29.6
	≥3log10	57	17.4

Duration of ART use (years)	<2	78	23.8
	2-5	93	28.4
	≥5	157	50.8

WHO clinical stage of the disease	Stage I	265	80.8
	Stage II	37	11.3
	Stage III or IV	26	7.9

**Table 4:** Associated factors of cognitive impairment among HIV-positive patients at JUMC, Jimma, Ethiopia, 2018 (N = 328).

*Variable*	Yes (n = 117)	No (n = 213)	COR (95% CI)	AOR (95% CI)
*Age (years)*				
18-30	21 (17.9%)	69 (32.7%)	1.0	1.0
31-40	34 (29.1%)	93 (44.1%)	1.2 (0.6,2.2)	1.2 (0.6,2.8)
41-64	62 (53%)	49 (23.2%)	4.1 (2.2,7.6)	*3.1 (1.3,7.4)*
*Educational status *				
≤Grade 8	82 (70.1%)	66 (31.3%)	6.9 (3.4,13.8)	1.2 (0.5,3.3)
Grade 9-12	23 (19.7%)	78 (37.0%)	1.6 (0.7,3.6)	2.5 (0.9,6.6)
Diploma and above	12 (10.3%)	67 (31.8%)	1.0	1.0
*Income (ETB/month)*				
<500	41 (35.0%)	27 (12.8%)	5.6 (2.9,10)	1.5 (0.53,4.0)
500-1000	43 (36.8%)	54 (25.6%)	2.9 (1.6,5.3)	2.2 (0.9,5.1)
1001-1500	9 (7.7%)	41 (19.4%)	0.8 (0.3,1.9)	0.5 (0.2,1.4)
≥1500	24 (20.5%)	89 (42.2%)	1.0	1.0
*Lifetime khat chewing *				
Yes	75 (66.4%)	75 (66.4%)	8.1 (4.8,13.6)	*4.4 (2.3,8.3)*
No	42 (19.5%)	42 (19.5%)	1.0	1.0
*Comorbid hypertension*				
Yes	54 (46.2%)	21 (10%)	7.75 (4.3,13.8)	2.2 (0.98,5.02)
No	63 (53.8%)	190 (90%)	1.0	1.0
*Plasma HIV 1 RNA load (copies/ml) *				
<1.7log10	35 (29.9%)	139 (65.9%)	1.0	1.0
1.7log10-3log10	35 (29.9%)	62 (29.4%)	2.2 (1.3,3.9)	*2.2 (1.1,4.3)*
≥3log10	47 (40.2%)	10 (4.7%)	18.6 (8.6,40.6)	*7.5 (2.6,21.5)*
*Stage of the disease *				
Stage I	76 (65%)	189 (89.6%)	1.0	1.0
Stage II	22 (18.8%)	15 (7.1%)	3.6 (1.79,7.4)	0.9 (0.3,2.7)
Stage III and above	19 (16.2%)	7 (3.3%)	6.8 (2.7,16.7)	*5.6 (1.7,19.2)*

*Note.* Numerical data in bold indicate statistical significance (p < 0.05). ETB = Ethiopian Birr; COR = crude odds ratio; AOR = adjusted odds ratio.

## Data Availability

The authors confirm that all data underlying the findings are fully available without restriction. All relevant data are within the manuscript.
